# Longitudinal Investigation of Enteric Virome Signatures from Parental-Generation to Offspring Pigs

**DOI:** 10.1128/spectrum.00023-23

**Published:** 2023-05-11

**Authors:** Qu Chen, Xiaojun Zhang, Weiling Shi, Xizhong Du, Lingyan Ma, Wen Wang, Shiyu Tao, Yingping Xiao

**Affiliations:** a State Key Laboratory for Managing Biotic and Chemical Threats to the Quality and Safety of Agro-products, Institute of Agro-product Safety and Nutrition, Zhejiang Academy of Agricultural Sciences, Hangzhou, China; b Institute of Animal Husbandry and Veterinary Medicine, Jinhua Academy of Agricultural Sciences, Jinhua, China; c Zhejiang Dovro Animal Health Business Company, Jinhua, China; d College of Animal Sciences and Technology, Huazhong Agricultural University, Wuhan, China; University of California, San Diego

**Keywords:** swine gut virome, metagenome, dynamics, auxiliary metabolic genes

## Abstract

To date, studies on the swine gut microbiome have focused almost exclusively on bacteria. Despite recent advances in the understanding of the swine gut bacteriome at different growth stages, a comprehensive longitudinal study of the lifetime dynamics of the swine gut virome is lacking. Here, we used metagenomic sequencing combined with bioinformatic analysis techniques to characterize the gut viromes of parental-generation and offspring pigs at different biological classification levels. We collected 54 fecal samples from 36 parental-generation pigs (18 breeding boars [Duroc] and 18 pregnant/lactating sows [Landrace]) and 108 fecal samples from 18 offspring pigs during the lactation (day 3), nursery (days 26, 35, and 49), growing (day 120), and finishing (day 180) stages. Alpha diversity, including community richness (richness index) and diversity (Shannon index), showed an overall increasing trend in offspring pigs. Distinct shifts (beta diversity) in the microbiome structure along different growth stages were observed. The linear discriminant analysis effect size (LEfSe) algorithm revealed 53 viral genus that are stage specific. Host prediction results showed that enteric viruses are probably correlated with carbohydrate decomposition. We identified abundant auxiliary carbohydrate-active enzyme (CAZyme) genes from enteric viruses, most of which are glycoside hydrolase genes and participate in the biolysis of complex polysaccharides.

**IMPORTANCE** This study shows that distinct stage-associated swine gut viromes may be determined by age and/or gut physiology at different growth stages, and enteric viruses probably manipulate carbohydrate decomposition by abundant glycoside hydrolases. These findings fill a gap in the longitudinal pattern of the swine gut virome and lay the foundation for research on the function of swine enteric viruses.

## INTRODUCTION

The swine gut microbiome is now thought to play an integral role in growth, health, and disease (1–3). Persistent alterations in the composition, diversity, and function of the gut microbiome are increasingly being recognized as key factors in the establishment and maintenance of a growing number of disease states (4), including diarrhea (5) and constipation (6). The gut microbiome consists of bacteria, viruses, fungi, and archaea. Within the gut microbial consortium, the bacteriome has attracted the most attention in the vast majority of studies (7–9). However, the important roles of viruses in maintaining host health and physiological functions are often ignored (10). Increasing evidence from studies suggests that the gut virome regulates metabolism, growth, and disease progression through various paths of interaction with the cooccurring bacteriome and directly with the host (11, 12).

The microbial community composition is dynamic and can develop from complex interplays between host and external environmental factors, including age, sex, and diet (13). The bacterial composition is by far the most investigated and has been shown to be highly temporally stable in healthy adults (14). Infants start their lives with a gut that is largely sterile (15), and prokaryotes colonize the intestinal tract step by step (16) until the age of ~2 years, when it reaches a stable adult-like composition (17). The assessment of the role of the gut virome is lagging; previous studies showed that the gut virome can be divided into eukaryotic viruses (viruses that infect eukaryotic cells, primarily human cells in the gut) and prokaryotic viruses (viruses that infect prokaryotic cells, mostly bacteria), of which prokaryotic viruses account for >90% of the viruses in the human gut (18). Eukaryotic RNA viruses are rare under healthy conditions, and most of them are reported to be plant viruses that may originate from diets (19, 20). Both adult and infant gut virota are dominated by bacteriophages, whereas RNA phages are significantly less abundant (18). Most bacteriophages have remained unidentified, complicating virome analyses (21, 22). With the wide application of high-throughput sequencing technologies, increasing numbers of studies are focusing on studying the structure and function of the gut microbiome of pigs (23, 24). However, longitudinal studies on the intestinal viral profile and function in pigs are still lacking.

The purpose of this study is to explore the longitudinal changes in the swine enteric virome from parental-generation to offspring pigs, covering the lactation, nursery, growing, and finishing stages. The existing evidence described above suggests that under physiological conditions, the influence of intestinal DNA viruses on the host is dominant; therefore, we examined DNA viruses in the feces of healthy pigs using metagenomic techniques. Furthermore, we performed a bioinformatic analysis of the viral profiles obtained by sequencing to reveal the structure and function of the viral communities in pigs. Our study is the first to systematically investigate the changes in the intestinal viral signatures and functions from parental-generation to offspring pigs, which will help fill the research gaps in the field and provide the foundation for further studies in the future.

## RESULTS

### Alpha and beta diversities of fecal viruses in parental-generation and offspring pigs.

**FIG 1:**
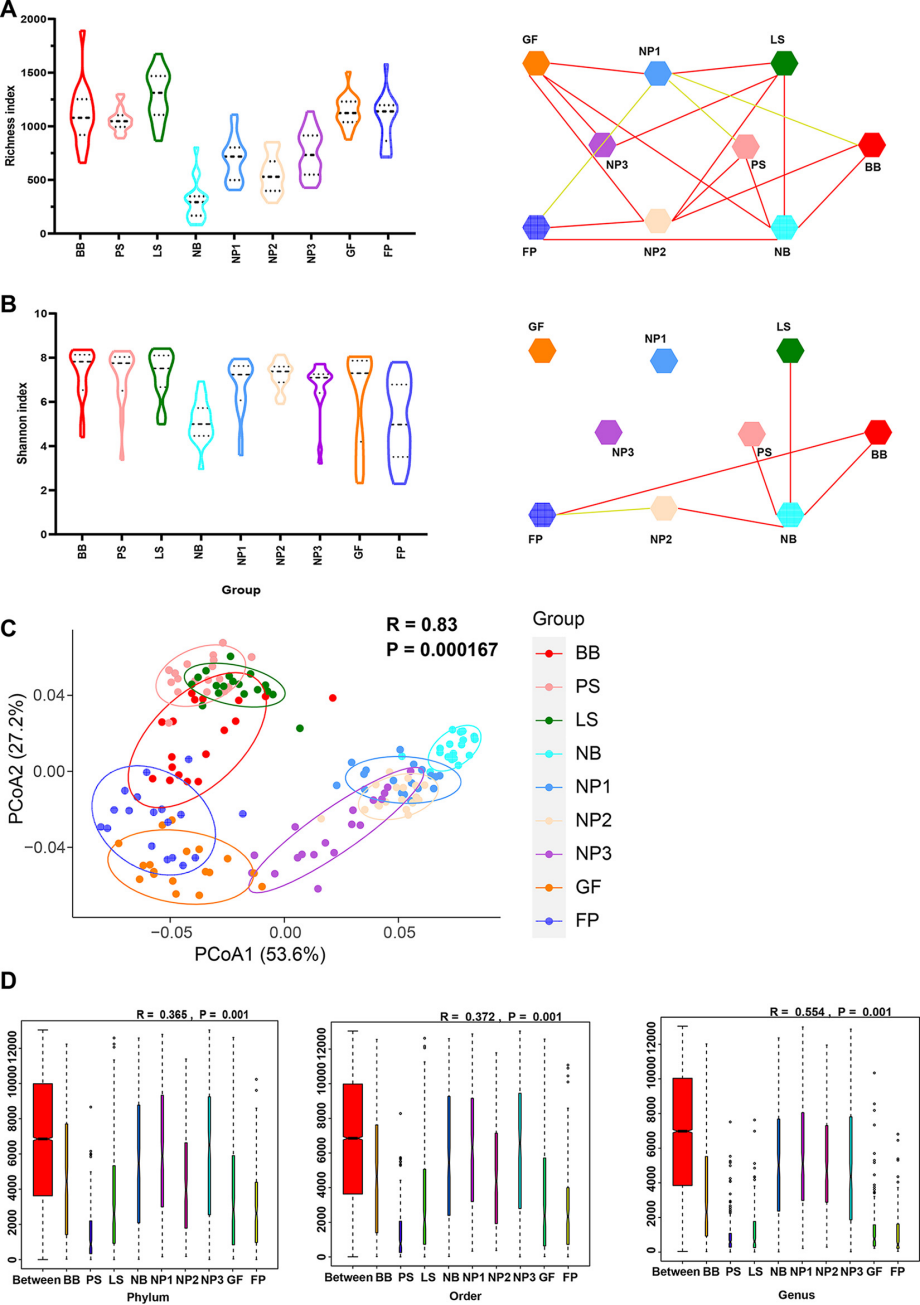
Characterization of the longitudinal changes in the swine gut virome. Richness indices (A), Shannon indices (B), PCoA based on Bray-Curtis distances (C), and analysis of similarities (ANOSIM) of intestinal viruses at the phylum, order, and genus levels (D) are shown. The connection between two nodes represents a significant difference. Significance was determined using the Kruskal-Wallis test and the *post hoc* Mann-Whitney U test. Red, *P < *0.01; yellow, *P < *0.05.

To investigate the changes in the alpha diversity of intestinal viruses in each group, we analyzed richness and Shannon indices at the read level. For parental-generation pigs, the richness indices were not significantly different among all groups (*P >* 0.05). For offspring pigs, the overall richness index increased over time, and starting from the birth stage, the richness in the NB, NP1, NP2, and NP3 groups was significantly lower than that in the parental-generation pigs (*P <* 0.05), while the richness in the GF and FP groups did not show a significant difference (*P > *0.05) (Fig. 1A). The changing trend of the Shannon index was similar to that of the richness index; the Shannon index in the NB group was significantly lower than that for the parental-generation pigs (*P < *0.05), while the NP1, NP2, NP3, and GF groups did not show a significant difference (*P > *0.05). However, the Shannon index decreased at the finishing stage, and the Shannon index in the FP group was lower than that in the BB group (*P < *0.05) (Fig. 1B). In general, the richness and Shannon indices increased with the growth of the piglets and gradually approximated those of the parental-generation pigs.

Next, we investigated the changes in the overall viral phenotypes in each group. Principal-coordinate analysis (PCoA) showed that the viral profiles of the parental-generation pigs were significantly different from those of the offspring pigs at the operational taxonomic unit (OTU) level. With the growth of the piglets, their viral profiles gradually became more similar to those of the parental-generation pigs (Fig. 1C). Analysis of similarity (ANOSIM) showed that viral signatures were significantly distinct between the parental-generation and offspring pigs (NB, NP1, NP2, and NP3) at the phylum, order, and genus levels (*P < *0.05) (Fig. 1D).

### Species composition of fecal viruses in parental-generation and offspring pigs.

**FIG 2:**
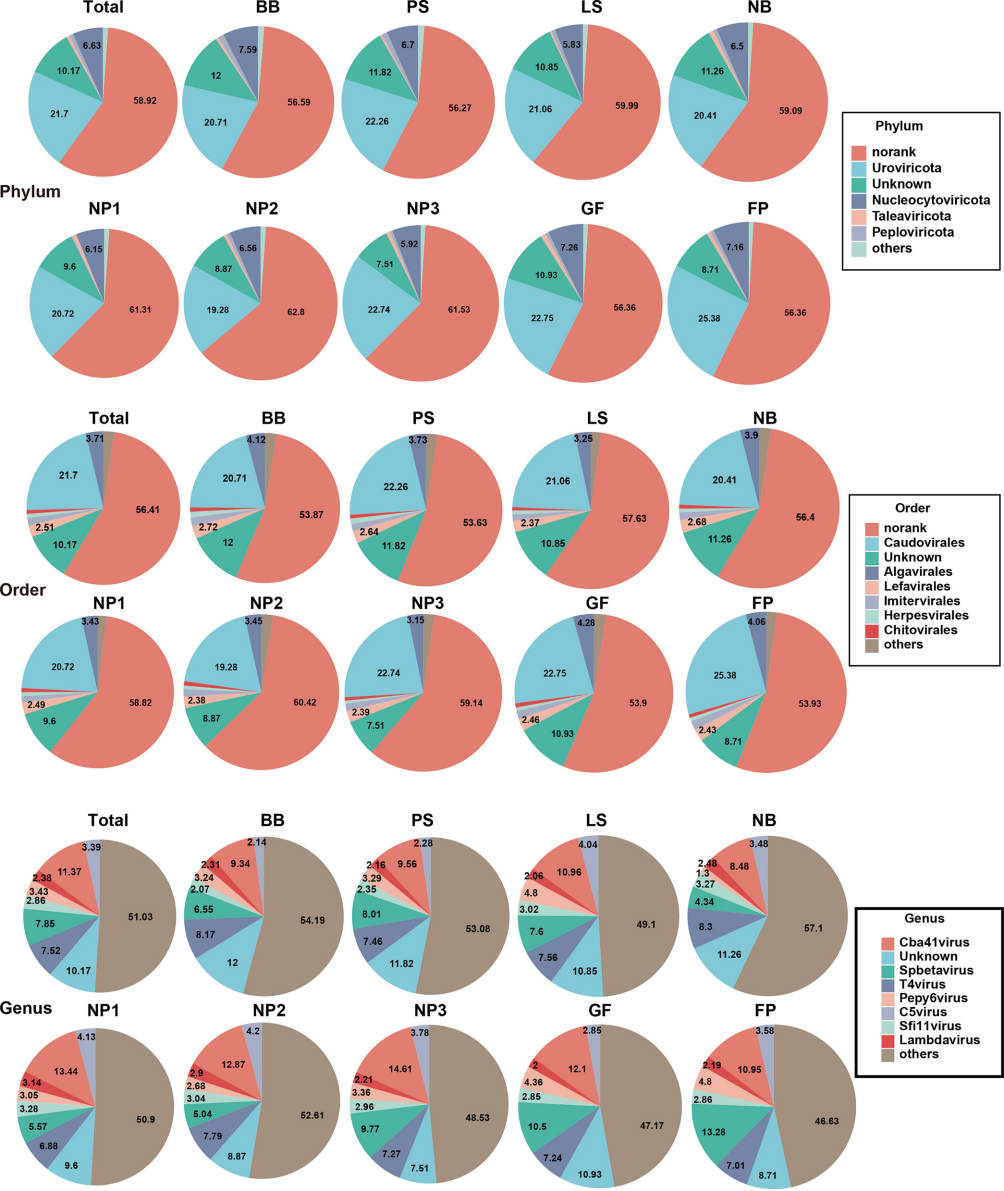
Community composition of the gut viromes in parental-generation and offspring pigs at the phylum, order, and genus levels.

The species compositions of viruses at the phylum, order, and genus levels are shown in Fig. 2. At the phylum level, more than half of the viral taxa were not classified (no rank), and *Uroviricota*, *Nucleocytoviricota*, *Preplasmiviricota*, and *Peploviricota* were the predominant viruses in the feces from all groups. The abundances of these viruses in each group remained relatively stable. At the order level, more than half of the viral taxa were not classified, and *Caudovirales*, *Algavirales*, *Lefavirales*, and *Imitervirales* were the predominant viruses in all groups. *Caudovirales* were increased in the FP group, *Algavirales* were decreased in the NP3 group, and *Lefavirales* remained stable in each group. At the genus level, the abundances of no-rank viruses decreased in the NP3, GF, and FP groups. *Spbetavirus*, *Cba41virus*, and *T4virus* were the predominant viruses in all groups. *Cba41virus* was the most frequently annotated virus in most groups and constituted more than 8.6% of all viruses, *Spbetavirus* was the most frequently annotated virus in the BB and FP groups, and *P12024virus* was the most frequently annotated virus in the NP2 group. Except for the NP2 group, *Spbetavirus* constituted more than 4.9% of all viruses. The abundance of *P12024virus* was high only in the NP2 and NP3 groups and was <1.86% in the other groups.

### The core and stage-associated viromes.

**FIG 3:**
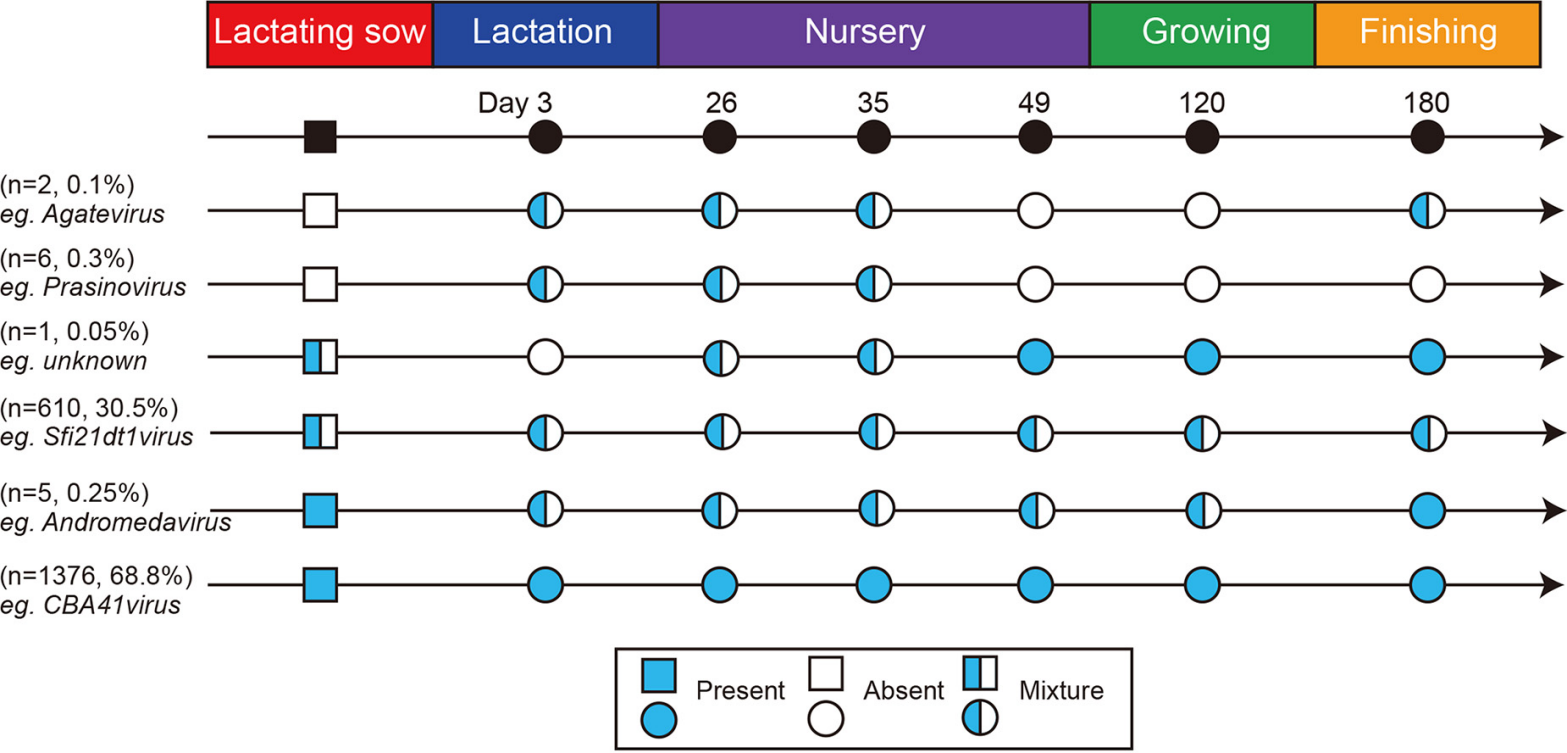
Longitudinal occurrence patterns of the swine gut virome. The top 2,000 OTUs based on average relative abundances in each group were used to summarize the occurrence patterns. Blue circles indicate the presence of a viral taxon, while white circles indicate its absence. Mixed-color circles indicate a transition between presence and absence in each group.

The top 2,000 OTUs based on average relative abundance were used to summarize the patterns of occurrence of swine gut viruses. The order and timing of the appearance of the swine gut virome members are shown in Fig. 3. Among the top 2,000 OTUs, 1,376 OTUs were present in all lactating sows and offspring pigs and are defined as the “core” virome or residents of the swine gastrointestinal (GI) tract. For example, *CBA41virus* was a resident that was observed in each individual among all groups. A total of 615 OTUs were present in all groups but not each individual, such as *Sfi21dt1virus* and *Andromedavirus*. OTUs that appeared sporadically at certain stages but disappeared later are called “passengers”; in our data, passengers appeared mainly at the lactation and nursery stages (6 OTUs) and occasionally at the finishing stage (2 OTUs). For example, *Prasinovirus* is a passenger that was observed only at the lactation and nursery stages. In addition, we did not observe OTUs that appeared only at certain stages; however, an unknown OTU disappeared only at the lactation stage, while it could be observed in lactating sows and weanling offspring pigs. These results suggested that the vast majority of major swine gut viruses may persist in pig herds through vertical transmission, with only a few likely to be affected by age or diet.

**FIG 4:**
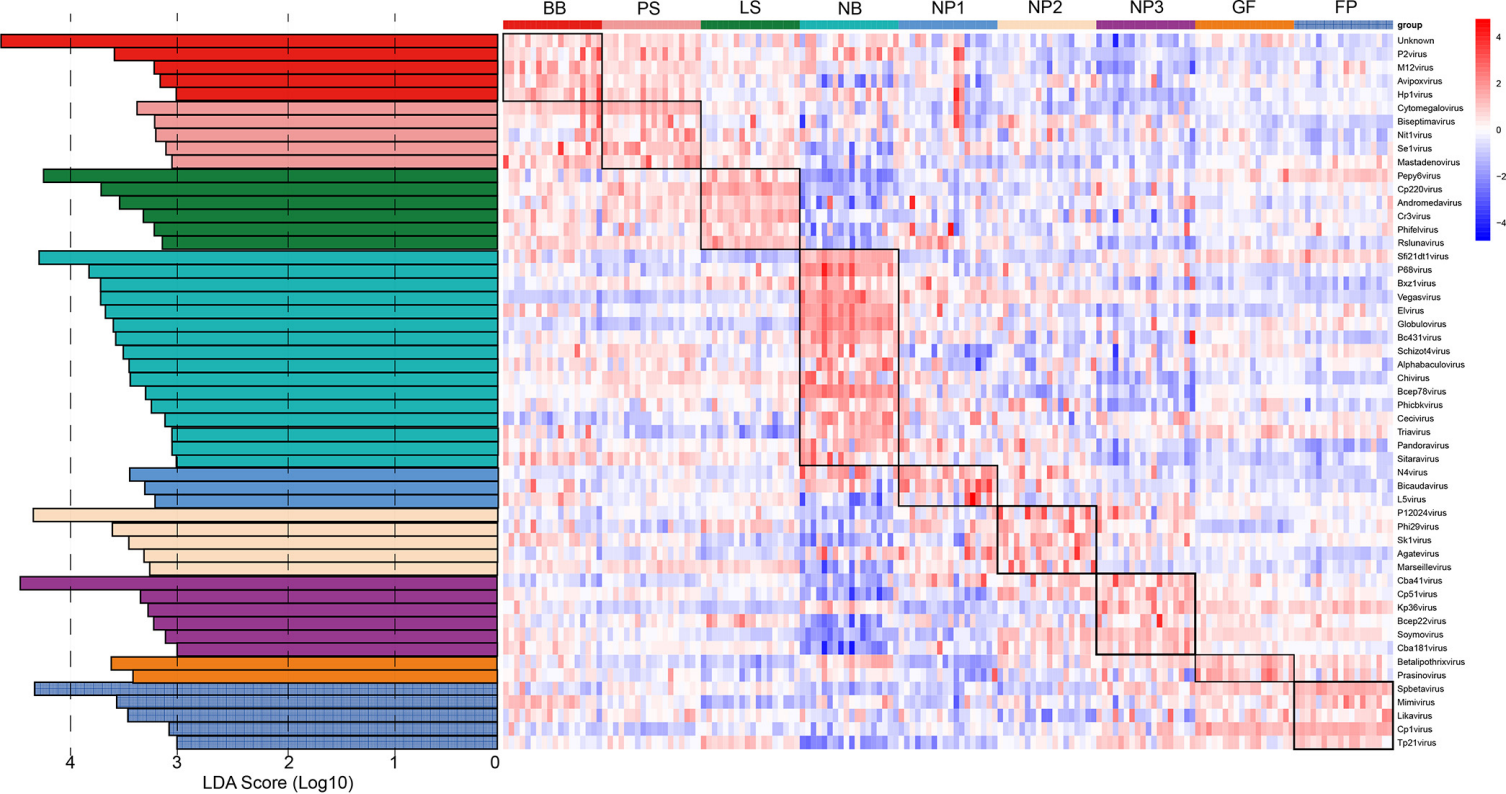
Heatmap showing characteristic viruses at the genus level identified by the LEfSe algorithm (LDA score of >3). The heatmap shows the average relative abundances on a log scale.

**FIG 5:**
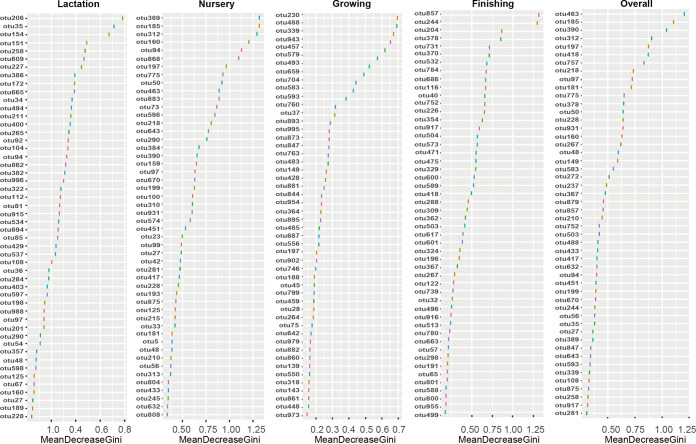
Characteristic viruses at each growth stage based on the random-forest algorithm. The top 50 characteristic viruses at the lactation, nursery, growing, finishing, and overall stages were selected from the top 1,000 OTUs (based on average relative abundance) using the regression-based random-forest algorithm in R. The abscissa represents the importance indicator value based on MeanDecreaseGini, and the ordinate represents the OTU sorted by MeanDecreaseGini. The higher the MeanReducedGini value, the higher the indicative value of the OTU.

The linear discriminant analysis (LDA) effect size (LEfSe) and random-forest algorithms were used to identify virus genus features (key viruses that distinguish differences between groups) in each group. The abundances of these features are visualized on a heatmap. As shown in Fig. 4, there were 5, 5, 6, 16, 3, 5, 6, 2, and 5 characteristic viral genera in the BB, PS, LS, NB, NP1, NP2, NP3, GF, and FP groups, respectively. The viruses enriched in the LS group had the lowest abundances in the NB group, indicating that the number of these viruses transmitted vertically is limited. The number of viruses enriched in the NB group was large, and these viruses decreased rapidly after weaning, indicating that these viruses have a vital role in milk digestion. Except for the NB group, the number of viruses enriched in the NP3 group was the largest, and these viruses were maintained at a relatively high abundance in the subsequent growth stages. In addition, the abundances of viruses enriched in the GF and FP groups were close to those in the parental-generation pigs, indicating that these viruses may be related to solid-feed digestion and growth. We next classified the enteric viral community (top 1,000 OTUs) based on the random-forest algorithm to distinguish the key viruses in pigs at different growth stages. The characteristic top 50 viral OTUs at each growth stage are listed in Fig. 5. For example, OTU206 (*C5virus*), OTU312 (*Cba41virus*), OTU230 (*T4virus*), and OTU244 (*Yatapoxvirus*) were found to be characteristic viruses at the lactation, nursery, growing, and finishing stages, respectively.

### Cooccurrence analysis of enteric viruses in different developmental stages.

**FIG 6:**
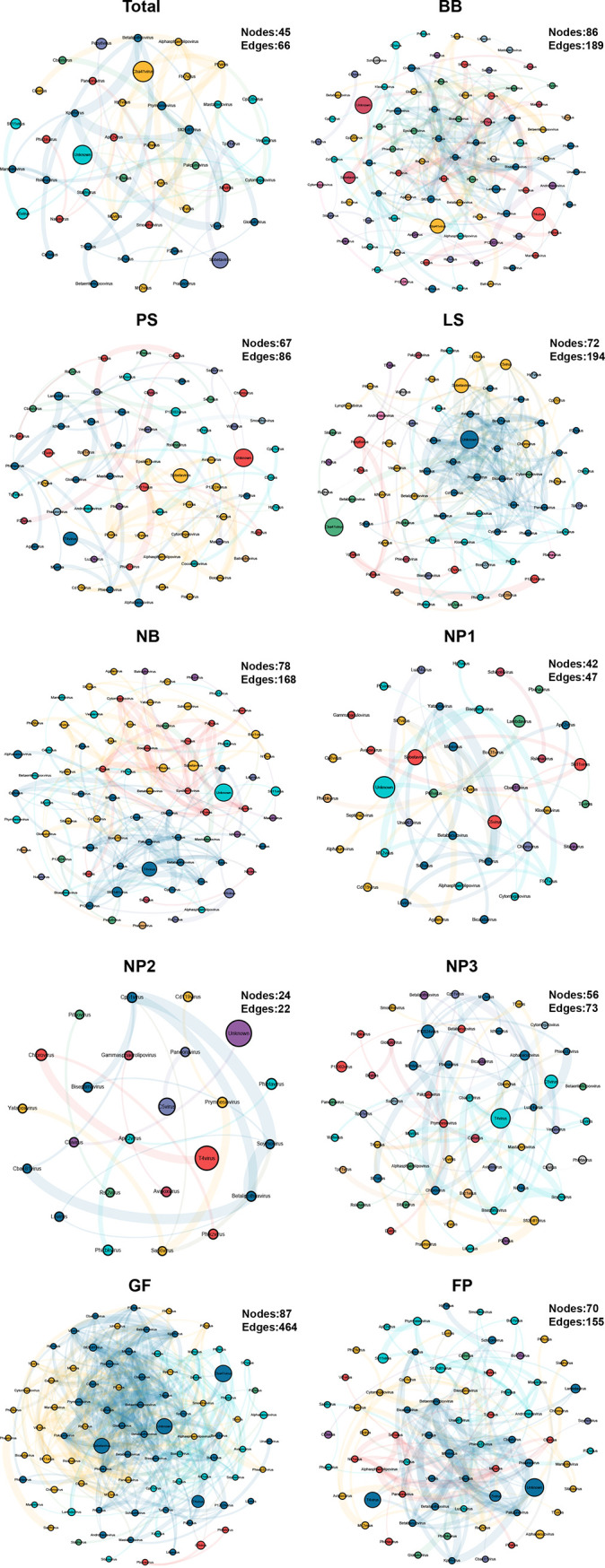
Cooccurrence network analysis of the interactions between viral taxa. Nodes represent different virus taxa, and edges represent correlations. The network diagram shows only significant correlations between viruses (Spearman correlation coefficient [|*r*|] of >0.6; *P* value of <0.05).

In order to investigate the overall differences in the gut viruses in pigs at different developmental stages, we performed a cooccurrence network analysis. The cooccurrence networks showed that the GF group had the highest complexity, consisting of 87 nodes and 464 edges, while the NP2 group had the lowest complexity, consisting of 24 nodes and 22 edges. The cooccurrence network complexity was dynamic, showing a decreasing-increasing-decreasing trend (Fig. 6).

**FIG 7:**
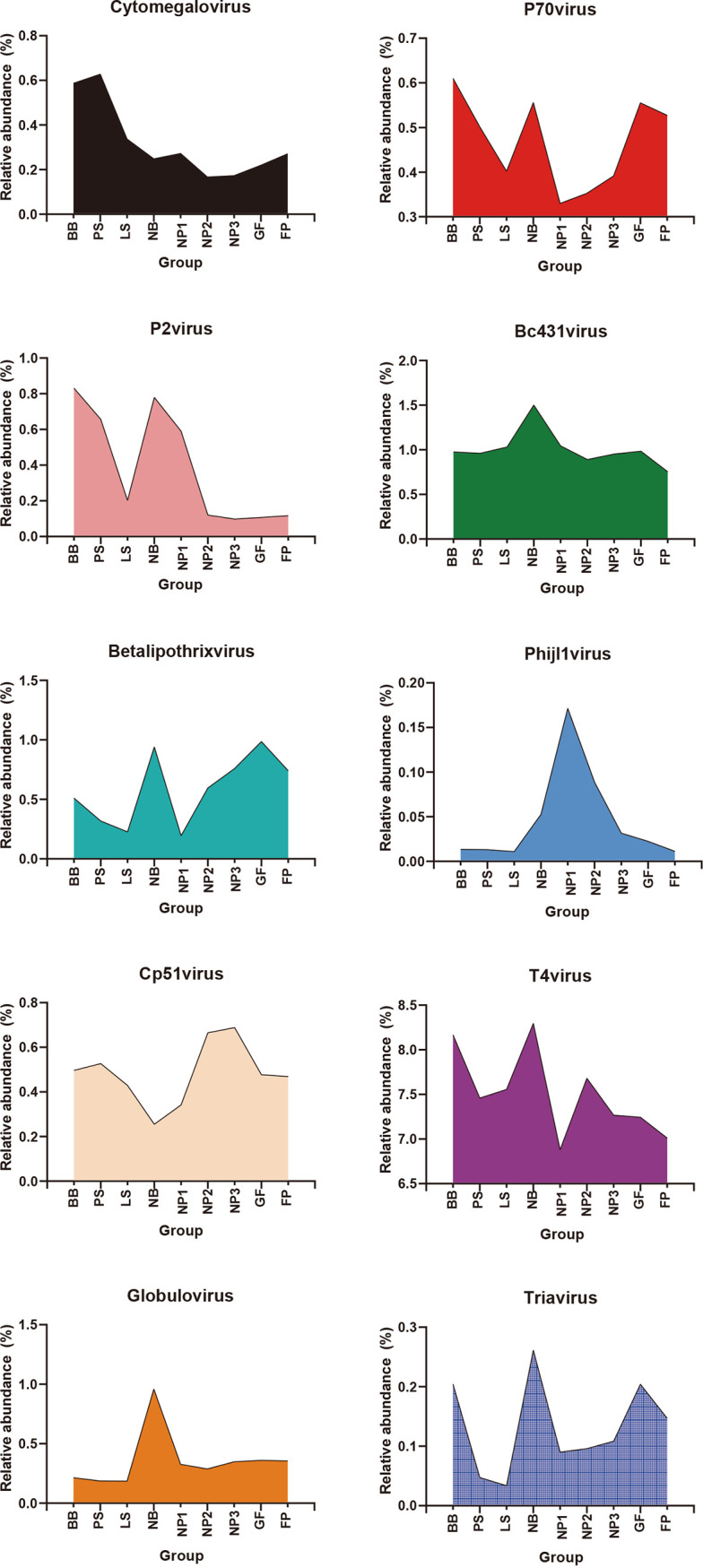
Dynamic changes in the relative abundances (percentages) of the core viruses with the highest degree centrality in each group.

Information on the key nodes of each group in cooccurrence networks is shown in Table 1, including degree centrality, betweenness centrality, and abundance. We selected the nodes with the highest degree centrality in each group to plot their dynamic changes (Fig. 7). We found that the abundances of these viruses changed dramatically during the nursery period; the abundances of *P70virus*, *P2virus*, *Bc431virus*, *Betalipothrixvirus*, *T4virus*, and *Globulovirus* were decreased, while the abundances of *Phijl1virus* and *Cp51virus* were increased. In the subsequent growth process, the abundances of *P2virus*, *Bc431virus*, *Betalipothrixvirus*, *T4virus*, and *Globulovirus* remained relatively stable, while the abundances of *P70virus*, *Cp51virus*, and *Triavirus* changed in the growing period.

**TABLE 1:** Core viruses of the cooccurrence network in each group

Group and virus	Degree	Betweenness	Abundance
Total			
Cytomegalovirus	10	88	0.336375
Sfi21dt1virus	7	28	1.499307
P2virus	7	2	0.406124
Alphasphaerolipovirus	7	35	0.295117
Hp1virus	7	10	0.240373
BB			
P70virus	18	391	0.634194
Bicaudavirus	14	54	0.30899
PS			
P2virus	9	371	0.681119
P1virus	8	414	0.331488
LS			
Unknown	21	164	11.27436
Bc431virus	17	180	1.071362
Prasinovirus	17	63	0.994875
NB			
Betalipothrixvirus	14	85	0.971243
Pakpunavirus	13	68	0.275404
NP1			
Phijl1virus	7	71	0.178397
Biseptimavirus	5	14	0.500332
Yatapoxvirus	5	31	0.206152
NP2			
Cp51virus	5	10	0.708573
Soymovirus	5	10	0.212498
NP3			
T4virus	7	71	7.546325
M12virus	6	330	0.610663
Biseptimavirus	6	111	0.483912
GF			
Globulovirus	26	92	0.374377
Cp8virus	25	168	1.11861
Betaentomopoxvirus	25	154	0.252947
Triavirus	25	116	0.212263
FP			
Triavirus	13	262	0.151442
Sfi21dt1virus	11	758	2.523482
Mimivirus	11	887	1.754858
Cbastvirus	11	447	0.562213
C5virus	11	200	3.692784

### Host prediction and carbohydrate metabolic gene annotations for enteric viruses.

**FIG 8:**
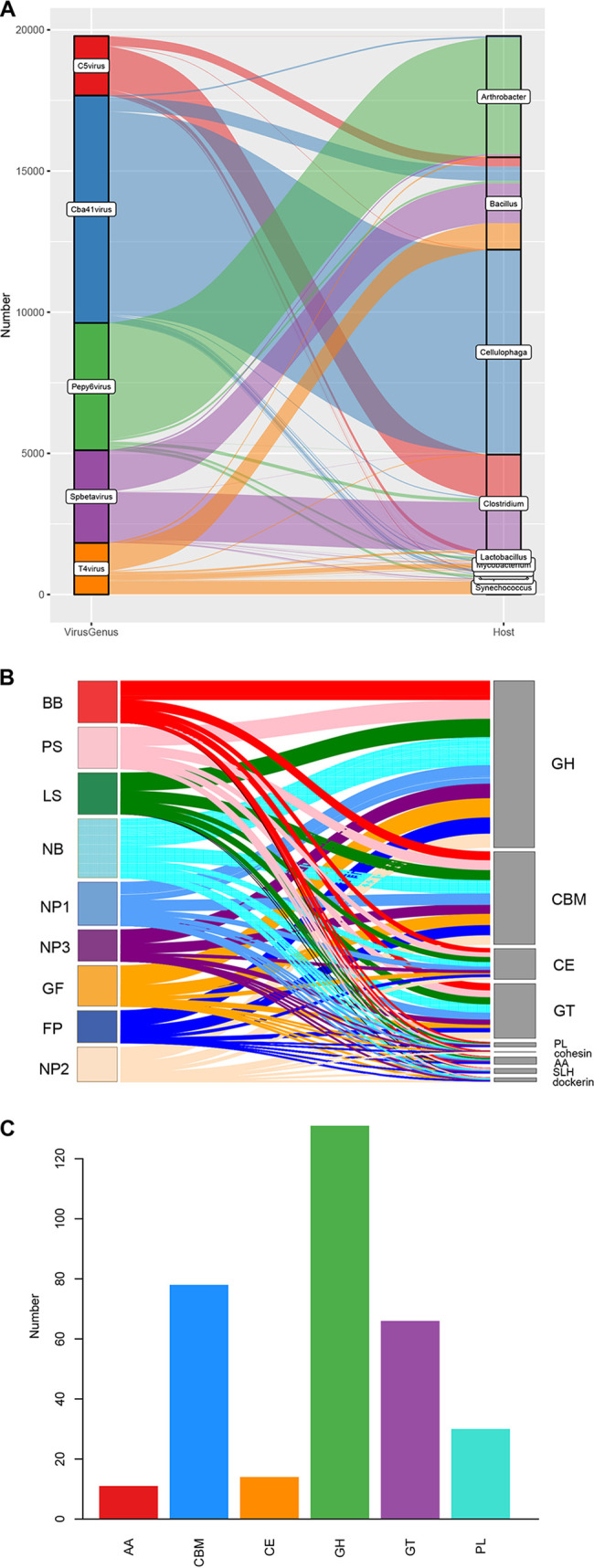
Virus host prediction and abundant auxiliary carbohydrate metabolism gene annotations. (A) Sankey plot showing the predicted hosts of the virus genera with the highest abundances (top 5). (B) Sankey plot showing the annotation of viral carbohydrate-metabolism-related unigenes in the CAZy database. (C) Number of types of annotated auxiliary CAZymes. GH, glycoside hydrolase; GT, glycosyltransferase; PL, polysaccharide lyase; CE, carbohydrate esterase; AA, auxiliary activity; CBM, carbohydrate-binding module.

We further predicted the hosts of the five enteric viruses with the highest abundances. As shown in Fig. 8A, most of the hosts of these viruses were predicted to be *Arthrobacter*, *Bacillus*, *Cellulophaga*, and *Clostridium*, and the remaining hosts were predicted to be mainly *Lactobacillus*, Mycobacterium, and *Synechococcus*. Most of the hosts of *C5virus* were predicted to be *Clostridium*, most of the hosts of *Cba41virus* were predicted to be *Cellulophaga*, most of the hosts of *Pepy6virus* were predicted to be *Arthrobacter*, most of the hosts of *Spbetavirus* were predicted to be *Clostridium*, and most of the hosts of *T4virus* were predicted to be *Bacillus*.

We further annotated viral carbohydrate-metabolism-related unigenes (VCUs) in the CAZy database by using the dbCAN server based on the recognition of the carbohydrate-active enzyme (CAZyme) signature domain. According to the results of CAZy database annotations, most of the VCUs were annotated as glycoside hydrolases (GHs), followed by carbohydrate-binding modules (CBMs) and glycosyl transferases (GTs). The numbers of VCUs annotated as GHs, CBMs, and GTs in the NB group were higher than those in the other groups (Fig. 8B). In addition, the numbers of types of viral auxiliary CAZymes annotated as GHs were also the highest, followed by CBMs and GTs (Fig. 8C).

## DISCUSSION

In recent years, increasing attention has been paid to research on human and animal enteric viromes. A recent study on the human gut microbiome constructed reference databases of DNA viruses by using an assembly metagenomic approach, and more than 75% of the genomes represented double-stranded DNA phages that infect members of the classes *Bacteroidia* and *Clostridia* (25). At present, most studies on swine enteric viruses have focused mainly on disease models (26, 27). In fact, as the main component of the gut microbiome, enteric viruses play an important role in maintaining intestinal homeostasis and physiological functions (28). For intestinal bacteria, researchers have long recognized that they may play an important role in the growth and development of pigs, and they found that several key bacteria are closely associated with growth performance (29). However, there is still no longitudinal investigation of the swine gut virome. Consistent with previous studies on gut bacteria (30, 31), an overall increasing trend in the alpha community richness and diversity of the gut virome was observed during the preharvest life span of the pigs. The richness and Shannon indices plateaued at the growing stage. The high alpha diversities at the growing and finishing stages were very comparable to those in the parental-generation pigs, indicating a fully developed swine gut virome before entry into the market. However, the changes in the gut virome during human infant development do not seem to be completely consistent with those of piglets. A longitudinal study of the structure of the infant gut virome revealed that bacterial, eukaryotic, and archaeal viruses were established in early life, and the richness and diversity of phages are greatest at birth, gradually reducing from birth to 2 years of age (32). An inverse relationship between phageome diversity and bacteriome diversity is present in the gut microbiome of human infants (32), while a consistent upward trend is present in piglets. These results suggest that the swine enteric virome may follow a developmental pattern different from that of humans.

This study provides a comprehensive view of the succession of the swine gut virome from birth to market by longitudinally collecting fecal samples from the same set of pigs across different growth stages. This helps us to understand the changes in the swine gut virome over time across different growth stages. PCoA plots based on Bray-Curtis distances showed distinct clusters completely separating the parental-generation and offspring pigs (except for the FP group). Moreover, the PCoA structure of the gut virome in piglets gradually became similar to that of parental-generation pigs, strictly followed by the development of growth stages. Interestingly, researchers observed dramatic changes in both the community structure and composition of the swine gut bacteriome at 7 days postweaning, which might be attributed to weaning stress and/or the introduction of solid food (29). However, in our study, weaning stress did not cause dramatic changes in the alpha and beta diversities of the swine enteric virome. Until the growing stage, the enteric virome of piglets showed significant differences and became similar to that of the parental-generation pigs. Therefore, compared to enteric bacteria, the overall composition of viruses is less sensitive to weanling stress. Similarly, a previous study found that probiotic supplementation did not change the enteric virome in piglets and that the general composition of the enteric virome was dependent mainly on age (33). In this study, through a longer experimental period, we further confirmed that the pig enteric virome is more likely to be affected by age than by diet. We speculated that the pig enteric virome may be affected by the bacteriome, which would result in the hysteresis of virome composition variation.

This study also enabled us to address the following important biological questions regarding the swine gut virome. (i) Which viral taxa are residents, persisting in the whole preharvest section? (ii) Which viruses are passengers, present at only a certain point in time? (iii) What are the origins of the swine gut virome (e.g., vertical transmission, diet, or environment)? At all biological taxonomic levels, viruses with the highest abundances in each group were highly similar (e.g., *Uroviricota*, *Nucleocytoviricota*, *Cba41virus*, and *T4virus*). These viruses were early colonizers of the swine gut and persisted throughout the preharvest life span, from the sow milk-based lactation stage to the solid-feed-based nursery, growing, and finishing stages, indicating that these vertically transmitted viruses played a leading role in maintaining the stability of the swine enteric virome. It is worth noting that these viruses accounted for the vast majority of swine enteric viruses, and 68.8% of the viruses existed in each individual. Only one new virus appeared and persisted after the introduction of solid feed, while 6 viruses disappeared after the introduction of solid feed. The similarity between the patterns of occurrence in the swine gut virome and bacteriome is because the majority of the viruses and bacteria persisted throughout the preharvest life span through vertical transmission. The difference is that the introduction of solid feed has a greater impact on enteric bacteria: more bacteria disappear, but new colonizers appear and persist (29). Interestingly, the cooccurrence network of the virome was altered dramatically after the introduction of solid feed. The complexity of the cooccurrence network did not recover until the late nursery stage. Moreover, most of the key viruses selected in the cooccurrence network analysis were also altered significantly at the nursery stage, suggesting that the nursery stage may be the critical period for the construction of the swine enteric virome.

According to the results of host predictions for the five viruses with the highest abundances, the most frequently annotated bacterium was *Cellulophaga*. It is worth mentioning that the LEfSe and random-forest algorithms showed that *Cba41virus* is a characteristic virus in the intestine of nursery piglets. *Cellulophaga* is Gram negative and can be found in soil and seawater; it is worth mentioning that it is very important for carbohydrate decomposition and can decompose one or more kinds of agar, alginic acid, cellulose, and chitin (34, 35). Interestingly, the functions of swine viral unigenes were annotated mainly to metabolic pathways in the Kyoto Encyclopedia of Genes and Genomes (KEGG) database (see Fig. S2 in the supplemental material). A previous study extensively investigated viral auxiliary metabolic genes (AMGs) in marine environments and found that most of them are involved in central carbon metabolism to promote viral replication (36). A previous study on bovine rumen viruses revealed that carbon AMGs were associated with carbohydrate metabolism; these viral AMGs contain five glycosidic hydrolases, including β-glucosidase, α-glucosidase, α-amylase, maltooligosyltrehalose, trehalohydrolase, and endoglucanase (37). In our study, four rumen virus-encoded glycosidic hydrolases (β-glucosidase, α-glucosidase, α-amylase, trehalohydrolase, and endoglucanase) were also identified in the swine gut virome. Furthermore, we identified more AMGs in the swine gut virome, such as peptidoglycan hydrolase, β-xylosidase, galactocerebrosidase, β-hexosaminidase, and chitinase (Table S1). This study is the first to report such viral AMGs in the swine gut virome. Interestingly, most viral carbohydrate metabolic genes encode CAZymes with glycoside hydrolase activities, suggesting that swine gut viruses may contribute to the decomposition of organic carbon. In addition, the number of viral unigenes annotated by the CAZy database changed significantly in nursery piglets, indicating that the introduction of solid fiber-rich feed during the nursery stage may change the metabolic function of swine enteric viruses.

### Conclusion.

To the best of our knowledge, our study is the first to perform a longitudinal investigation of the characteristics of the enteric virome in parental-generation and offspring pigs by metagenomic techniques. We found that the overall structure of the gut virome of piglets during the lactation and nursery stages was different from that of parental-generation pigs. With increasing age, the viral composition of the offspring gradually became similar to that of the parental generations. The dominant swine enteric viruses came from vertical transmission and persisted stably throughout the preharvest life span. Nursery stages may be the key periods for the transformation of the gut virome in piglets. Remarkably, swine gut viruses were related to metabolic functions, and we identified abundant auxiliary CAZyme genes from them, which were involved primarily in the decomposition of organic carbon. Collectively, our results show that swine enteric viruses may follow a unique developmental pattern different from that of swine enteric bacteria or human enteric viruses. Moreover, the important and diverse metabolic functions of swine enteric viruses may be greater than previously suspected.

## MATERIALS AND METHODS

### Ethics statement, experimental design, and sample collection.

**FIG 9:**
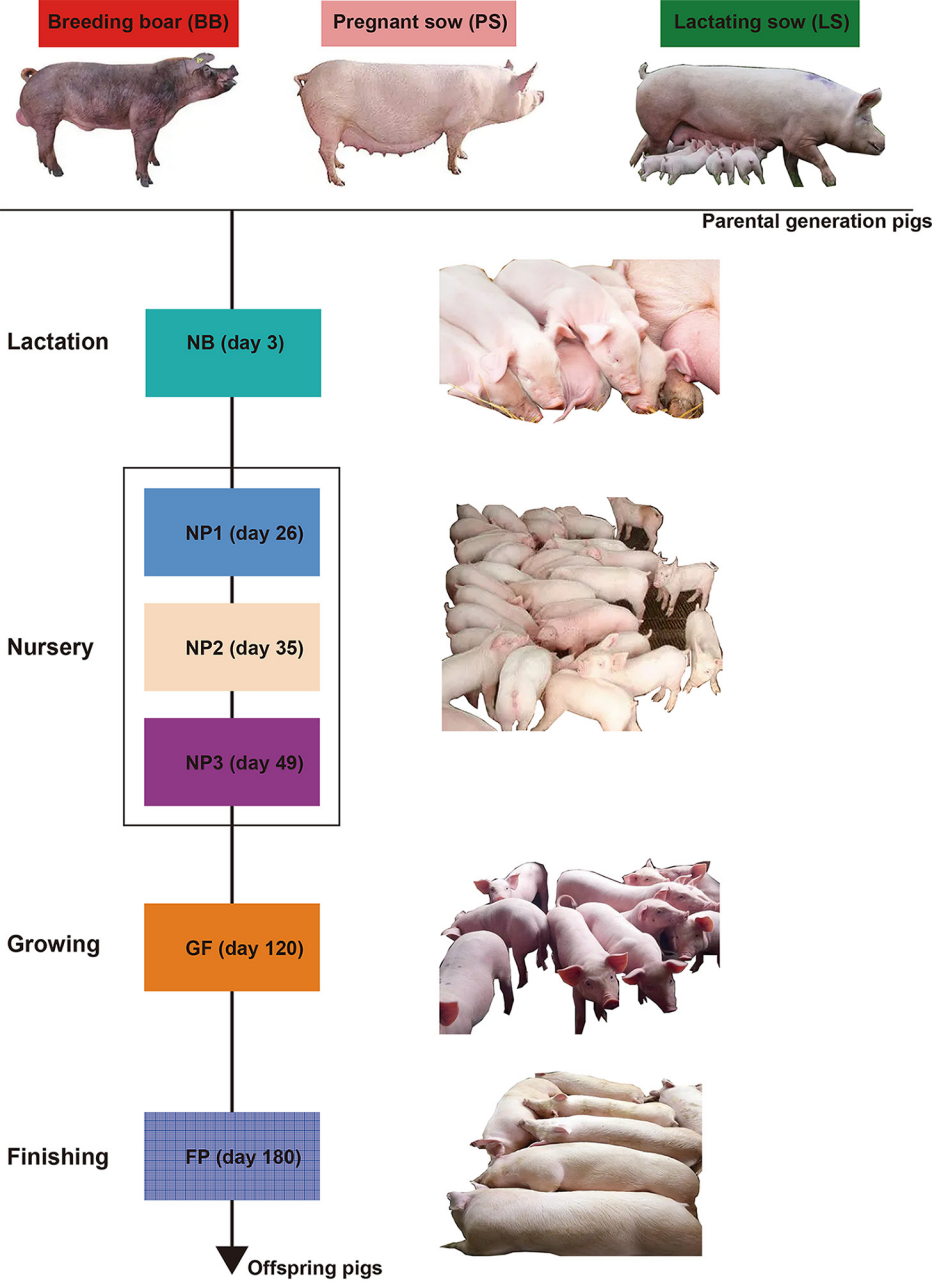
Experimental design.

All animal experiments were approved by the Institutional Animal Care and Use Committee of the Zhejiang Academy of Agricultural Sciences in accordance with the relevant rules and regulations (ZAAS-2017-009). Fifty-four fecal samples from 36 parental-generation pigs (18 breeding boars [BB] [Duroc] and 18 pregnant sows [PS]/lactating sows [LS] [Landrace]) and 108 fecal samples from 18 male offspring pigs during the lactation (day 3 [NB]), nursery (day 26 [NP1], day 35 [NP2], and day 49 [NP3]), growing (day 120 [GF]), and finishing (day 180 [FP]) stages were collected and used for metavirome sequencing. The experimental design is shown in Fig. 9. All collected fecal samples were immediately frozen in liquid nitrogen and stored at −80°C.

### DNA extraction, library preparation, and high-throughput sequencing.

According to the manufacturer’s protocols, total DNA was extracted from 250-mg fecal samples using the E.Z.N.A. viral DNA kit (Omega Bio-Tek, Norcross, GA, USA). High-quality DNA samples (optical density at 260/280 nm [OD_260/280_] of ~1.8 to 2.2 and OD_260/230_ of ≥2.0) were used to construct the sequencing library. The DNA was amplified before library preparation. Metagenomic libraries were prepared using the TruSeq Nano DNA sample preparation kit from Illumina (San Diego, CA), using 1 μg of total DNA. Illumina’s protocol was followed for DNA end repair, A-base addition, and the ligation of Illumina-indexed adaptors. The libraries were sized using 2% low-range ultra-agarose for selecting DNA target fragments of ~400 bp, followed by PCR amplification using Phusion DNA polymerase (New England BioLabs [NEB]) for 15 cycles. Metagenomic sequencing was performed by Shanghai Biozeron Biotechnology Co., Ltd. (Shanghai, China). All samples were sequenced in paired-end 150-bp mode on a next-generation sequencing platform.

### Read quality control and mapping.

The raw paired-end reads were trimmed and quality controlled by Trimmomatic version 0.40 (38) with the parameters SLIDINGWINDOW:4:15 MINLEN:75 (http://www.usadellab.org/cms/?page=trimmomatic). Next, clean reads that aligned to the pig genome (NCBI assembly accession number GCF_000003025.6) were also removed. Further analysis was carried out on this set of high-quality reads.

### Read-based phylogenetic annotation.

The taxonomy of clean reads for each sample was determined with Kraken2 (39) using the customized kraken database. The customized kraken database included all virus genome sequences in the NCBI RefSeq database (release 204). All reads were classified to the domain, phylum, class, order, family, genus, species, and unclassified levels. Bracken (https://ccb.jhu.edu/software/bracken/) was used to estimate the abundances of taxonomy; it can produce accurate species- and genus-level abundances even in multiple nearly identical species. The relative abundance of a certain level is the total abundance of species belonging to a certain level.

### *De novo* assembly, gene prediction, and annotation.

Clean sequence reads generated a set of contigs for each sample using MegaHit with the parameters –min-contig-len 500 (40). Assembly sequences of >2,000 bp were selected for the assembled genome, and VirFinder (41), VirSorter2 (42), CAT (43), and the IMG/VR (Integrated Microbial Genomes) database (https://img.jgi.doe.gov/cgi-bin/vr/main.cgi) were used in parallel for virus identification of these sequences.

Mummer software was used to cluster vOTUs (viral operational taxonomic units) as candidate virus sequences with more than 95% similarity and 85% coverage of the total sequence length, and the longest representative one within each cluster was considered a vOTU (44). Virus species annotation and host prediction were performed using the VPF-Class tool (45).

METAProdigal (46) (http://compbio.ornl.gov/prodigal/) was used to predict all of the genes, and the genes were then searched against the NCBI protein nonredundant (NR), String, eggNOG (47), and Kyoto Encyclopedia of Genes and Genomes (KEGG) databases using BLASTX to identify the proteins that had the highest sequence similarity with the given transcripts to retrieve their functional annotations, and a typical cutoff E value of <1.0 × 10^−5^ was set. Quality-filtered sequences were annotated by the Comprehensive Antibiotic Resistance Database (CARD) (https://card.mcmaster.ca/). For the CAZymes, we first predicted the open reading frames of the contigs using Prodigal in metagenomic mode (48). Next, CAZymes were annotated using hmmscan against custom CAZyme families built from dbCAN2 and Pfam with a cutoff E value of 1 × 10^−15^ and a coverage of 0.35 (49). The functional abundances were normalized to the total annotated sequences.

### Swine enteric virome data analysis.

Alpha diversity (richness and Shannon index) determinations were conducted and visualized using the tidyverse (version 1.3.1) and vegan (version 2.5-7) packages in R (https://www.r-project.org/), respectively. The differences in alpha diversity between two groups were identified using the Wilcoxon rank sum test, and *P* values of <0.05 were considered statistically significant. Principal-coordinate analysis (PCoA) plots based on Bray-Curtis distances were used to visualize the differences in viral communities and functional traits. Permutational multivariate analysis of variance (PERMANOVA) was used to perform analyses of the differences between groups (50). Analysis of similarity (ANOSIM) was also conducted in qiime2 (version 2.4). Viral features differentially represented in each group were identified by using the linear discriminant analysis (LDA) effect size (LEfSe) algorithm (LDA score of >3) (51). The cooccurrence network was calculated on the basis of relative abundances with Spearman’s rank correlation coefficient (*P < *0.05) using the R package Hmisc (version 4.5-0). Cytoscape (version 3.8.2) software (52) was used to calculate and visualize the network layout. Only edges with correlations of >0.6 are displayed in the two nodes.
