# Supplementary material for: Longitudinal Investigation of Enteric Virome Signatures from Parental-Generation to Offspring Pigs
Source: Microbiol Spectr. 2023 May 11;11(3):e00023-23. doi: 10.1128/spectrum.00023-23 (PMC10269631; doi:10.1128/spectrum.00023-23)
Supplement: Supplemental file 3 — Legends to Fig. S1 and S2. Download spectrum.00023-23-s0001.pdf, PDF file, 0.2 MB [file spectrum.00023-23-s0001.pdf]

FIG S1 Composition of intestinal viruses in parental generation to offspring pigs at the phylum (A), order (B), and genus (C) levels.

FIG S2 Kyoto Encyclopedia of Genes and Genomes (KEGG) annotation of swine enteric virome.

Table 1 Core viruses of cooccurrence network in each group

Viruses	Degree	Betweenness	Abundance
Total			
Cytomegalovirus	10	88	0.336375
Sfi21dt1virus	7	28	1.499307
P2virus	7	2	0.406124
Alphasphaerolipovirus	7	35	0.295117
Hp1virus	7	10	0.240373
BB			
P70virus	18	391	0.634194
Bicaudavirus	14	54	0.30899
PS			
P2virus	9	371	0.681119
P1virus	8	414	0.331488
LS			
Unknown	21	164	11.27436
Bc431virus	17	180	1.071362
Prasinovirus	17	63	0.994875
NB			
Betalipothrixvirus	14	85	0.971243
Pakpunavirus	13	68	0.275404
NP1			
Phijl1virus	7	71	0.178397
Biseptimavirus	5	14	0.500332
Yatapoxvirus	5	31	0.206152
NP2			
Cp51virus	5	10	0.708573
Soymovirus	5	10	0.212498
NP3			
T4virus	7	71	7.546325
M12virus	6	330	0.610663
Biseptimavirus	6	111	0.483912
GF			
Globulovirus	26	92	0.374377
Cp8virus	25	168	1.11861
Betaentomopoxvirus	25	154	0.252947
Triavirus	25	116	0.212263
FP			
Triavirus	13	262	0.151442
Sfi21dt1virus	11	758	2.523482
Mimivirus	11	887	1.754858
Cbastvirus	11	447	0.562213
C5virus	11	200	3.692784

References

1. Su W, Gong T, Jiang Z, Lu Z, Wang Y. 2022. The Role of Probiotics in Alleviating Postweaning Diarrhea in Piglets From the Perspective of Intestinal Barriers. Front Cell Infect Microbiol 12:883107.
2. Duarte ME, Kim SW. 2022. Intestinal microbiota and its interaction to intestinal health in nursery pigs. Anim Nutr 8:169-184.
3. Wang H, Xu R, Zhang H, Su Y, Zhu W. 2020. Swine gut microbiota and its interaction with host nutrient metabolism. Anim Nutr 6:410-420.
4. Rose EC, Blikslager AT, Ziegler AL. 2022. Porcine Models of the Intestinal Microbiota: The Translational Key to Understanding How Gut Commensals Contribute to Gastrointestinal Disease. Front Vet Sci 9:834598.
5. Ren W, Yu B, Yu J, Zheng P, Huang Z, Luo J, Mao X, He J, Yan H, Wu J, Chen D, Luo Y. 2022. Lower abundance of Bacteroides and metabolic dysfunction are highly associated with the post-weaning diarrhea in piglets. Sci China Life Sci 65:2062-2075.
6. Yu X, Fu C, Cui Z, Chen G, Xu Y, Yang C. 2021. Inulin and isomalto-oligosaccharide alleviate constipation and improve reproductive performance by modulating motility-related hormones, short-chain fatty acids, and feces microflora in pregnant sows. J Anim Sci 99.
7. Newsome RC, Yang Y, Jobin C. 2022. The microbiome, gastrointestinal cancer, and immunotherapy. J Gastroenterol Hepatol 37:263-272.
8. Kadosh E, Snir-Alkalay I, Venkatachalam A, May S, Lasry A, Elyada E, Zinger A, Shaham M, Vaalani G, Mernberger M, Stiewe T, Pikarsky E, Oren M, Ben-Neriah Y. 2020. The gut microbiome switches mutant p53 from tumour-suppressive to oncogenic. Nature 586:133-138.
9. Paik D, Yao L, Zhang Y, Bae S, D'Agostino GD, Zhang M, Kim E, Franzosa EA, Avila-Pacheco J, Bisanz JE, Rakowski CK, Vlamakis H, Xavier RJ, Turnbaugh PJ, Longman RS, Krout MR, Clish CB, Rastinejad F, Huttenhower C, Huh JR, Devlin AS. 2022. Human gut bacteria produce TauEta17-modulating bile acid metabolites. Nature 603:907-912.
10. Aggarwala V, Liang G, Bushman FD. 2017. Viral communities of the human gut: metagenomic analysis of composition and dynamics. Mob DNA 8:12.
11. Keen EC, Dantas G. 2018. Close Encounters of Three Kinds: Bacteriophages, Commensal Bacteria, and Host Immunity. Trends Microbiol 26:943-954.
12. Yuan L, Hensley C, Mahsoub HM, Ramesh AK, Zhou P. 2020. Microbiota in viral infection and disease in humans and farm animals. Prog Mol Biol Transl Sci 171:15-60.
13. Hollister EB, Gao C, Versalovic J. 2014. Compositional and functional features of the gastrointestinal microbiome and their effects on human health. Gastroenterology 146:1449-58.
14. Poyet M, Groussin M, Gibbons SM, Avila-Pacheco J, Jiang X, Kearney SM, Perrotta AR, Berdy B, Zhao S, Lieberman TD, Swanson PK, Smith M, Roesemann S, Alexander JE, Rich SA, Livny J, Vlamakis H, Clish C, Bullock K, Deik A, Scott J, Pierce KA, Xavier RJ, Alm EJ. 2019. A library of human gut bacterial isolates paired with longitudinal multiomics data enables mechanistic microbiome research. Nat Med 25:1442-1452.
15. Perez-Munoz ME, Arrieta MC, Ramer-Tait AE, Walter J. 2017. A critical assessment of the "sterile womb" and "in utero colonization" hypotheses: implications for research on the

pioneer infant microbiome. Microbiome 5:48.
16. Stewart CJ, Ajami NJ, O'Brien JL, Hutchinson DS, Smith DP, Wong MC, Ross MC, Lloyd RE, Doddapaneni H, Metcalf GA, Muzny D, Gibbs RA, Vatanen T, Huttenhower C, Xavier RJ, Rewers M, Hagopian W, Toppari J, Ziegler AG, She JX, Akolkar B, Lernmark A, Hyoty H, Vehik K, Krischer JP, Petrosino JF. 2018. Temporal development of the gut microbiome in early childhood from the TEDDY study. Nature 562:583-588.
17. Yatsunenko T, Rey FE, Manary MJ, Trehan I, Dominguez-Bello MG, Contreras M, Magris M, Hidalgo G, Baldassano RN, Anokhin AP, Heath AC, Warner B, Reeder J, Kuczynski J, Caporaso JG, Lozupone CA, Lauber C, Clemente JC, Knights D, Knight R, Gordon JI. 2012. Human gut microbiome viewed across age and geography. Nature 486:222-7.
18. Shkoporov AN, Hill C. 2019. Bacteriophages of the Human Gut: The "Known Unknown" of the Microbiome. Cell Host Microbe 25:195-209.
19. Liang G, Bushman FD. 2021. The human virome: assembly, composition and host interactions. Nat Rev Microbiol 19:514-527.
20. Zhang T, Breitbart M, Lee WH, Run JQ, Wei CL, Soh SW, Hibberd ML, Liu ET, Rohwer F, Ruan Y. 2006. RNA viral community in human feces: prevalence of plant pathogenic viruses. PLoS Biol 4:e3.
21. Roux S, Hallam SJ, Woyke T, Sullivan MB. 2015. Viral dark matter and virus-host interactions resolved from publicly available microbial genomes. Elife 4.
22. Beller L, Matthijnssens J. 2019. What is (not) known about the dynamics of the human gut virome in health and disease. Curr Opin Virol 37:52-57.
23. Frese SA, Parker K, Calvert CC, Mills DA. 2015. Diet shapes the gut microbiome of pigs during nursing and weaning. Microbiome 3:28.
24. Guevarra RB, Hong SH, Cho JH, Kim BR, Shin J, Lee JH, Kang BN, Kim YH, Wattanaphansak S, Isaacson RE, Song M, Kim HB. 2018. The dynamics of the piglet gut microbiome during the weaning transition in association with health and nutrition. J Anim Sci Biotechnol 9:54.
25. Bolger AM, Lohse M, Usadel B. 2014. Trimmomatic: a flexible trimmer for Illumina sequence data. Bioinformatics 30:2114-20.
26. Wood DE, Salzberg SL. 2014. Kraken: ultrafast metagenomic sequence classification using exact alignments. Genome Biol 15:R46.
27. Li D, Liu CM, Luo R, Sadakane K, Lam TW. 2015. MEGAHIT: an ultra-fast single-node solution for large and complex metagenomics assembly via succinct de Bruijn graph. Bioinformatics 31:1674-6.
28. Ren J, Ahlgren NA, Lu YY, Fuhrman JA, Sun F. 2017. VirFinder: a novel k-mer based tool for identifying viral sequences from assembled metagenomic data. Microbiome 5:69.
29. Guo J, Bolduc B, Zayed AA, Varsani A, Dominguez-Huerta G, Delmont TO, Pratama AA, Gazitua MC, Vik D, Sullivan MB, Roux S. 2021. VirSorter2: a multi-classifier, expert-guided approach to detect diverse DNA and RNA viruses. Microbiome 9:37.
30. von Meijenfeldt FAB, Arkhipova K, Cambuy DD, Coutinho FH, Dutilh BE. 2019. Robust taxonomic classification of uncharted microbial sequences and bins with CAT and BAT. Genome Biol 20:217.
31. Roux S, Adriaenssens EM, Dutilh BE, Koonin EV, Kropinski AM, Krupovic M, Kuhn JH, Lavigne R, Brister JR, Varsani A, Amid C, Aziz RK, Bordenstein SR, Bork P, Breitbart M,

Cochrane GR, Daly RA, Desnues C, Duhaime MB, Emerson JB, Enault F, Fuhrman JA, Hingamp P, Hugenholtz P, Hurwitz BL, Ivanova NN, Labonte JM, Lee KB, Malmstrom RR, Martinez-Garcia M, Mizrachi IK, Ogata H, Paez-Espino D, Petit MA, Putonti C, Rattei T, Reyes A, Rodriguez-Valera F, Rosario K, Schriml L, Schulz F, Steward GF, Sullivan MB, Sunagawa S, Suttle CA, Temperton B, Tringe SG, Thurber RV, Webster NS, Whiteson KL, et al. 2019. Minimum Information about an Uncultivated Virus Genome (MIUViG). Nat Biotechnol 37:29-37.

32. Pons JC, Paez-Espino D, Riera G, Ivanova N, Kyrpides NC, Llabres M. 2021. VPF-Class: Taxonomic assignment and host prediction of uncultivated viruses based on viral protein families. Bioinformatics doi:10.1093/bioinformatics/btab026.

33. Hyatt D, LoCascio PF, Hauser LJ, Uberbacher EC. 2012. Gene and translation initiation site prediction in metagenomic sequences. Bioinformatics 28:2223-30.

34. Huerta-Cepas J, Szklarczyk D, Forslund K, Cook H, Heller D, Walter MC, Rattei T, Mende DR, Sunagawa S, Kuhn M, Jensen LJ, von Mering C, Bork P. 2016. eggNOG 4.5: a hierarchical orthology framework with improved functional annotations for eukaryotic, prokaryotic and viral sequences. Nucleic Acids Res 44:D286-93.

35. Hyatt D, Chen GL, Locascio PF, Land ML, Larimer FW, Hauser LJ. 2010. Prodigal: prokaryotic gene recognition and translation initiation site identification. BMC Bioinformatics 11:119.

36. Zhang H, Yohe T, Huang L, Entwistle S, Wu P, Yang Z, Busk PK, Xu Y, Yin Y. 2018. dbCAN2: a meta server for automated carbohydrate-active enzyme annotation. Nucleic Acids Res 46:W95-W101.

37. Doan T, Hinterwirth A, Worden L, Arzika AM, Maliki R, Abdou A, Kane S, Zhong L, Cummings SL, Sakar S, Chen C, Cook C, Lebas E, Chow ED, Nachamkin I, Porco TC, Keenan JD, Lietman TM. 2019. Gut microbiome alteration in MORDOR I: a community-randomized trial of mass azithromycin distribution. Nat Med 25:1370-1376.

38. Segata N, Izard J, Waldron L, Gevers D, Miropolsky L, Garrett WS, Huttenhower C. 2011. Metagenomic biomarker discovery and explanation. Genome Biol 12:R60.

39. Yang J, Zheng P, Li Y, Wu J, Tan X, Zhou J, Sun Z, Chen X, Zhang G, Zhang H, Huang Y, Chai T, Duan J, Liang W, Yin B, Lai J, Huang T, Du Y, Zhang P, Jiang J, Xi C, Wu L, Lu J, Mou T, Xu Y, Perry SW, Wong ML, Licinio J, Hu S, Wang G, Xie P. 2020. Landscapes of bacterial and metabolic signatures and their interaction in major depressive disorders. Sci Adv 6.

40. Nayfach S, Paez-Espino D, Call L, Low SJ, Sberro H, Ivanova NN, Proal AD, Fischbach MA, Bhatt AS, Hugenholtz P, Kyrpides NC. 2021. Metagenomic compendium of 189,680 DNA viruses from the human gut microbiome. Nat Microbiol 6:960-970.

41. Nantel-Fortier N, Gauthier M, L'Homme Y, Lachapelle V, Fravalo P, Brassard J. 2022. The swine enteric virome in a commercial production system and its association with neonatal diarrhea. Vet Microbiol 266:109366.

42. Tao S, Zou H, Li J, Wei H. 2022. Landscapes of Enteric Virome Signatures in Early-Weaned Piglets. Microbiol Spectr 10:e0169822.

43. Cao Z, Sugimura N, Burgermeister E, Ebert MP, Zuo T, Lan P. 2022. The gut virome: A new microbiome component in health and disease. EBioMedicine 81:104113.

44. Wang X, Tsai T, Deng F, Wei X, Chai J, Knapp J, Apple J, Maxwell CV, Lee JA, Li Y, Zhao

J. 2019. Longitudinal investigation of the swine gut microbiome from birth to market reveals stage and growth performance associated bacteria. Microbiome 7:109.
45. Lu D, Tiezzi F, Schillebeeckx C, McNulty NP, Schwab C, Shull C, Maltecca C. 2018. Host contributes to longitudinal diversity of fecal microbiota in swine selected for lean growth. Microbiome 6:4.
46. Chen L, Xu Y, Chen X, Fang C, Zhao L, Chen F. 2017. The Maturing Development of Gut Microbiota in Commercial Piglets during the Weaning Transition. Front Microbiol 8:1688.
47. Lim ES, Zhou Y, Zhao G, Bauer IK, Droit L, Ndao IM, Warner BB, Tarr PI, Wang D, Holtz LR. 2015. Early life dynamics of the human gut virome and bacterial microbiome in infants. Nat Med 21:1228-34.
48. Sachsenroder J, Twardziok SO, Scheuch M, Johne R. 2014. The general composition of the faecal virome of pigs depends on age, but not on feeding with a probiotic bacterium. PLoS One 9:e88888.
49. Gong Z, Liang Y, Wang M, Jiang Y, Yang Q, Xia J, Zhou X, You S, Gao C, Wang J, He J, Shao H, McMinn A. 2018. Viral Diversity and Its Relationship With Environmental Factors at the Surface and Deep Sea of Prydz Bay, Antarctica. Front Microbiol 9:2981.
50. Lisov AV, Kiselev SS, Trubitsina LI, Belova OV, Andreeva-Kovalevskaya ZI, Trubitsin IV, Shushkova TV, Leontievsky AA. 2022. Multifunctional Enzyme with Endoglucanase and Alginase/Glucuronan Lyase Activities from Bacterium Cellulophaga lytica. Biochemistry (Mosc) 87:617-627.
51. Hurwitz BL, U'Ren JM. 2016. Viral metabolic reprogramming in marine ecosystems. Curr Opin Microbiol 31:161-168.
52. Anderson CL, Sullivan MB, Fernando SC. 2017. Dietary energy drives the dynamic response of bovine rumen viral communities. Microbiome 5:155.
