# Supplementary figures and images for: Longitudinal Investigation of Enteric Virome Signatures from Parental-Generation to Offspring Pigs
Source: Microbiol Spectr. 2023 May 11;11(3):e00023-23. doi: 10.1128/spectrum.00023-23 (PMC10269631; doi:10.1128/spectrum.00023-23)

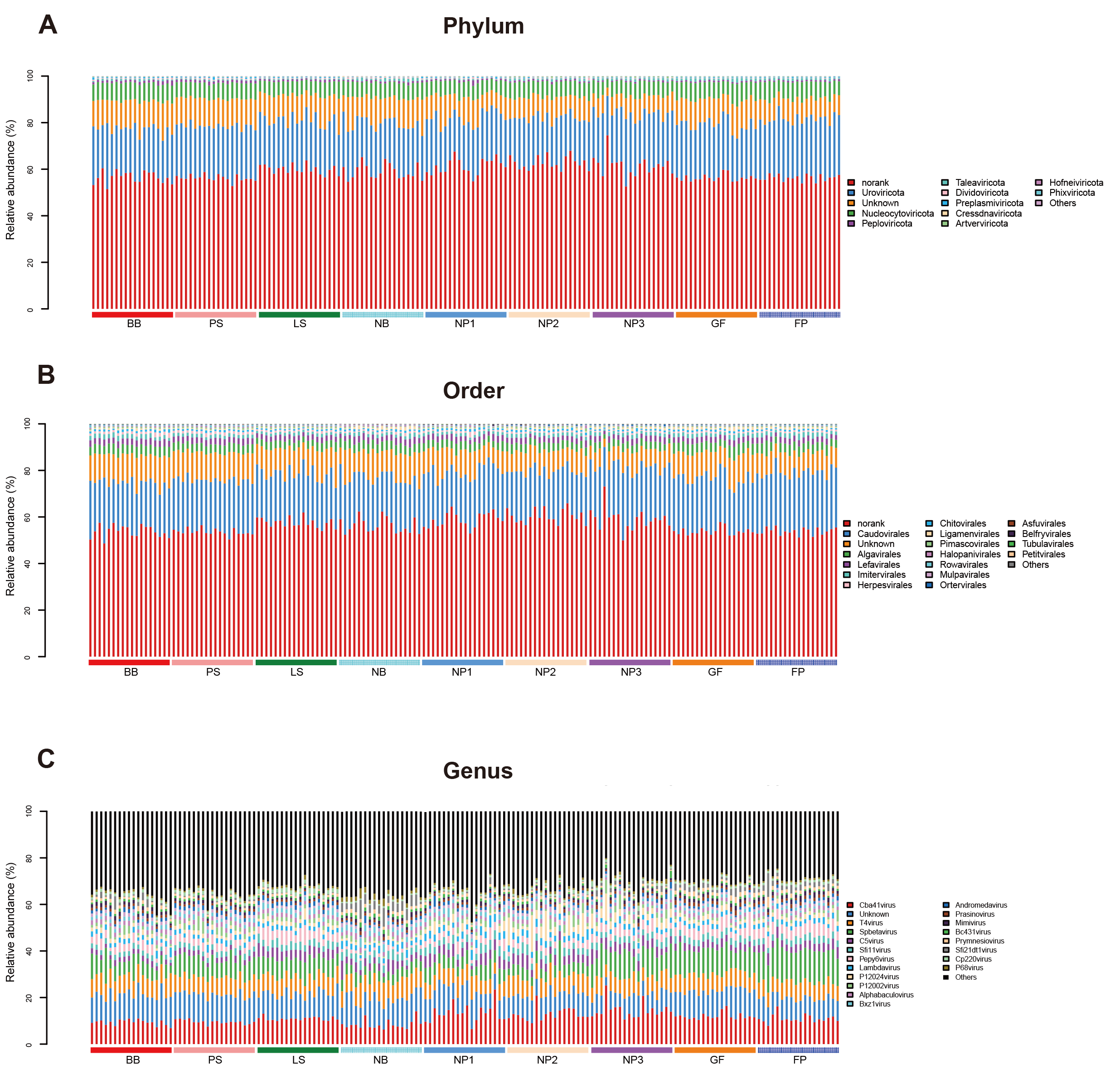

Supplement: Supplemental file 1 — Fig. S1. Download spectrum.00023-23-s0002.tif, TIF file, 2.0 MB [file spectrum.00023-23-s0002.tif]

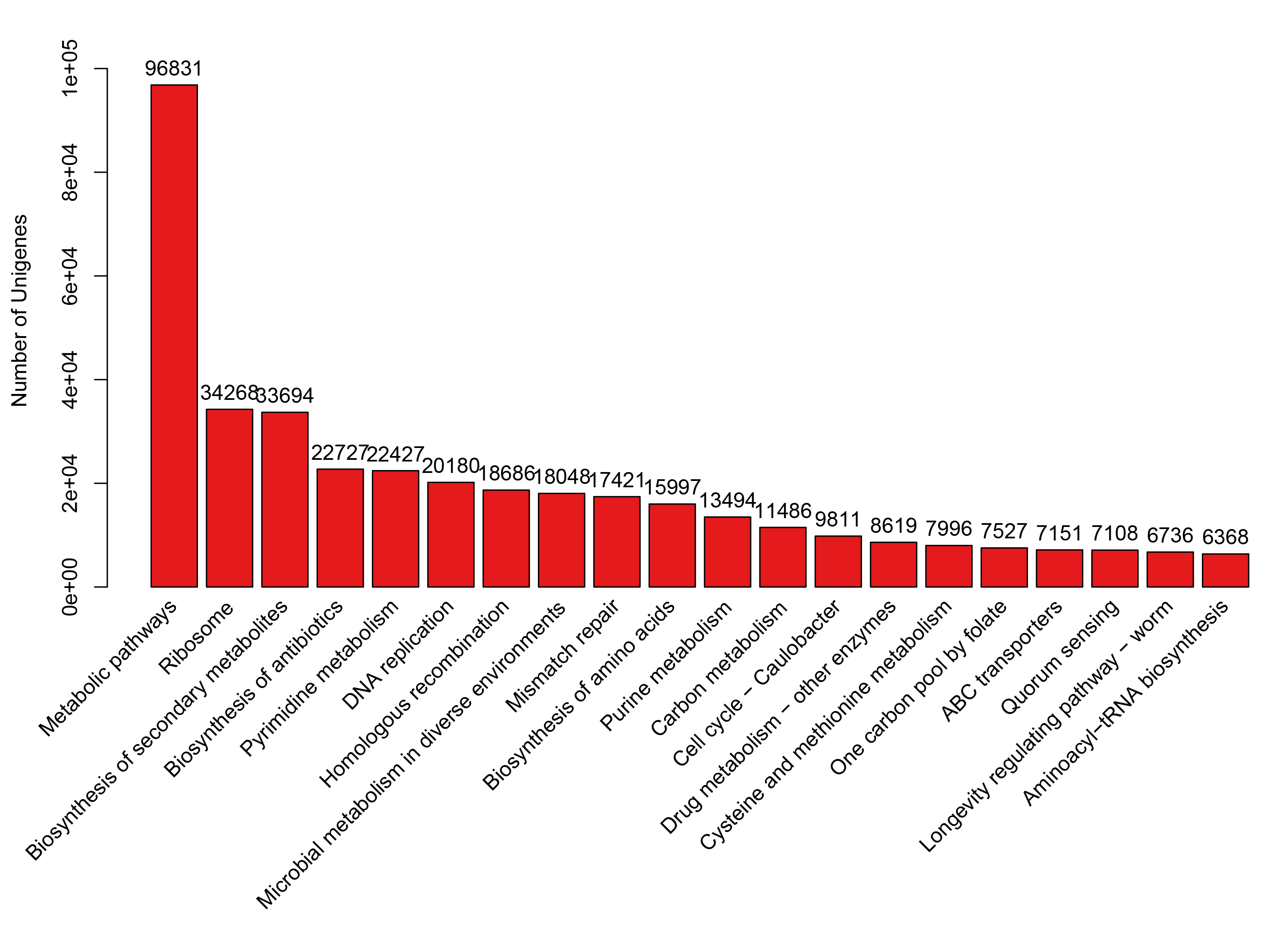

Supplement: Supplemental file 2 — Fig. S2. Download spectrum.00023-23-s0003.tif, TIF file, 0.3 MB [file spectrum.00023-23-s0003.tif]
